# Supplementary material for: Cardiovascular disease prevention: Community Based Asset Mapping within religious networks in a rural Sub-Saharan African neighbourhood
Source: PLOS Glob Public Health. 2023 Oct 20;3(10):e0002201. doi: 10.1371/journal.pgph.0002201 (PMC10588837; doi:10.1371/journal.pgph.0002201)
Supplement: S1 Data — (DOCX) [file pgph.0002201.s002.docx]

**Name:** c Public and private entities influencing health

<Files\\FGDs\\FGD-Female congregation members-01> - § 3 references coded [5.11% Coverage]

Reference 1 - 1.60% Coverage

I: What are the key public and private entities that influence health and well-being among people in your area?

R7: Navrongo health research center is one of them. As you are here, you want to screen people for diabetes and hypertension. Not only this, they go to the communities to educate people about diseases and how to practice personal hygiene to overcome such diseases.

R1: The social media also helps a lot especially the radio stations. If there are outbreaks of certain diseases, they announce and educate people about those conditions so that they know what to do to overcome such conditions.

Reference 2 - 1.66% Coverage

R2: I think the church is one of the organizations that help in health-related matters. When there is an outbreak of any disease or problem, they announce it in the church for others to hear because latterly not all people listen to radio and all that and they will enforce the congregants to strictly observe and obey everything that the health professional will instruct them to do. For example; when covid set in, most of the churches placed notices on the gates saying no mask, no entry. So, that way when you are coming to church, you will be compelled to wear a mask. So, I think they contribute to health and well-being.

Reference 3 - 1.85% Coverage

R8: The local authorities like the chiefs also help in the prevention of diseases. Sometimes the health facilities rely on them to organize health programmes for them.

R5: social media like WhatsApp, Facebook and the rest also give us information on the outbreak of diseases and how to prevent them.

R6: Community health providers. We used to see them come around to educate people on health issues including vaccines for the different age groups depending on who qualifies to take them. They will normally educate you on the health benefits and encourage you to take it. When covid came in, I saw a lot of people going round and educating people on the vaccine and encouraging them to take it.

<Files\\FGDs\\FGD-Male congregation members-07> - § 5 references coded [3.35% Coverage]

Reference 1 - 0.46% Coverage

R12: People do come, but it has been long since such people came and do research about health. They only come to ask about women and their children but not the general public.

Reference 2 - 0.79% Coverage

R5: What I can remember that made people come to our communities was when the COVID was at its peak. That was when people came to sensitize about it and showed us how we should protect ourselves from it. Apart from that, the people that also come is Navrongo research workers. That is what I also know.

Reference 3 - 0.70% Coverage

R9: What is also added is when the Ebola came. That time people use to come and sensitize us on how to protect ourselves. When the Ebola issue went down, we do not see them coming anymore but when the Ebola was at its peak killing people, they do come and talk to us.

Reference 4 - 0.92% Coverage

R11: What I observe is that, I see people that say they are VAST and come to ask us about our health. They come to our households and enquire about our health and the diseases that are affecting us. But this year, I haven't seen them yet. But they use to come and ask us about that or if there is any pregnant woman or any new person in the household.

Reference 5 - 0.47% Coverage

R2: In addition, we also see health workers come to the schools to talk to the teachers and the students in terms of health. That is what I have also observed and wanted to add.

<Files\\IDIs\\IDI-Elder- 01> - § 2 references coded [1.46% Coverage]

Reference 1 - 0.91% Coverage

I: What are the key public and private entities/organizations that influence health and wellbeing in your area?

R: I think that is the hospitals and clinics but there are also traditional people who are also in the system. They do advertisements in the radios and televisions.

Reference 2 - 0.55% Coverage

I: Can you mention names of the organizations that work to influence health in your area?

R: I only know of the ministry of health. The other practitioners are many.

<Files\\IDIs\\IDI-Elder-02> - § 1 reference coded [0.46% Coverage]

Reference 1 - 0.46% Coverage

R: For that one, it should be the CHPS compounds that are around, because we have two or three nurse that sometimes do talk about health.

<Files\\IDIs\\IDI-Elder-03> - § 2 references coded [2.74% Coverage]

Reference 1 - 2.08% Coverage

R: In this area as I know, the Ghana Health Service is there and once in a while they come and do education in the church. They normally write and inform us formally. Either they give to us to announce or they come themselves and do it. For instance, this year, they wrote to us about two or three times and they spoke about malaria. They had malaria vaccine given to children between the ages of zero to five years or so. So, the health sector is here helping us. Anytime they call on us, we collaborate with them and give them they, opportunity to do whatever they want to do to ensure our protection and good health. For now, I only know of the Ghana Health service. There was some one company like that which wanted to come and talk about diabetes. They wrote to us but at the end, they didn’t come. The name of the company is “LIANT” or something like that, I can’t even remember well. They wrote to us formally and announced to the people but at the end of the day, they didn’t come.

Reference 2 - 0.66% Coverage

R: Apart the private clinics such as wisdom clinic which is giving health services, we also have Wedam clinic, they were at the border but now they have moved to opposite the air strip. So, they are also helping and improving the health of the people. So those are the entities or the organizations that I know.

<Files\\IDIs\\IDI-Faith Leader-08> - § 1 reference coded [0.63% Coverage]

Reference 1 - 0.63% Coverage

R. Apart from the church, Research is doing well. They are making impact in our communities because their workers are always on the people when it comes to vaccines, they are helping us. They have been talking to our people on how they should live.

<Files\\IDIs\\IDI-Faith Nurse-10> - § 1 reference coded [3.51% Coverage]

Reference 1 - 3.51% Coverage

I: So, in this area that the church is located, do you know any organization that work to influence health in this area?

R: No. What I know is that the health workers at the war memorial hospital will normally come to do education on the cervical cancer and if you are interested, you go there for the screening. This doesn’t pertain to hypertension and diabetes. For hypertension, someone like me wanted to start it but I don’t have time. If I get people to support me, then we will do it. Hypertension and diabetes are not something that the people are born with and so if they knew what to do about it, they would’ve prevented it.

<Files\\IDIs\\IDI-Faith Nurse-12> - § 1 reference coded [0.64% Coverage]

Reference 1 - 0.64% Coverage

M: So, what in particular did they do to influence the health and wellbeing of the people?

R: They screened the people and then gave them education. Those that they screened, they screened for hepatitis A, B and C. So, if you had the C, they would help you but if you had the B then they helped you in treating it.

<Files\\IDIs\\IDI-Faith Nurse-15> - § 1 reference coded [1.64% Coverage]

Reference 1 - 1.64% Coverage

R: Yes, that one is there because when the health people came, there was no place for them, it was the church that gave them space to operate. First, we gave them this structure and later we said that since this is a block building and we don’t use it, why don’t we give it to them to help the community? This was when we then gave them the structure they are working in now.

I: So, as you said, this is a CHPS compound?

R: Yes, it is called the Saboro CHPS compound.

<Files\\IDIs\\IDI-Women Group Leader-17> - § 2 references coded [1.42% Coverage]

Reference 1 - 0.81% Coverage

R: To be frank, the government gave us health workers and also a place where they will be working in but in that place, I think they only monitor BP and related things but they do not have treatment over there. We also go for weighing over there.

Reference 2 - 0.62% Coverage

R: What I can also say is that some men or women do come and give malaria drugs to us to be giving to our children. They also give them some liquid drugs; they say it is sweet like toffee.

<Files\\IDIs\\IDI-Youth Leader-16> - § 3 references coded [2.35% Coverage]

Reference 1 - 0.70% Coverage

R. Like the health facility, sometimes the Nurses come around to speak to us. Sometimes on personal hygiene and then this COVID for us to know how to prevent the COVID and also personal hygiene; how you keep yourself clean. So, the Nurses sometimes do come around to have an education with us like we are doing

Reference 2 - 0.62% Coverage

M. So the Nurses that you talked about, is it the church that invites them or they come by themselves?

R. Sometimes the church invites them and sometimes they come by themselves too. We always have something that we call blue cross, I do not know whether you know about it

Reference 3 - 1.03% Coverage

R. Yes blue cross, it is a blue cross week. That one is about drugs abuse. So, we invite the Nurses to come and educate all members of the church on the effects of drugs abuse, what alcohol would do to your body when you take it rampantly and then what maybe cigarette or Indian hemp would do to your body. So blue cross week, we also invite the Nurses to come and then educate members of the church. So, from Monday to Friday the Nurses would always be here

<Files\\IDIs\\IDI-Youth Leader-18> - § 2 references coded [3.81% Coverage]

Reference 1 - 1.37% Coverage

I: Are there organizations that work to influence health positively in your community?

R: Apart from the research people that execute projects aimed at improving the health of people, I haven’t seen any other organization. The herbalists also come round from time to time to sell their products.

Reference 2 - 2.44% Coverage

I: Can you mention the things that the research has done to influence health?

R: The research people have a project that they have recruited children from the community and they usually check on them and carry out tests on them. Sometimes this helps and also these screening exercises that have been going on, I ever heard of one being carried out by the research center. It helps the people to know their health status. Besides, there are other projects that I may not remember that they do to advance the health of people.
