# Supplementary material for: Cardiovascular disease prevention: Community Based Asset Mapping within religious networks in a rural Sub-Saharan African neighbourhood
Source: PLOS Glob Public Health. 2023 Oct 20;3(10):e0002201. doi: 10.1371/journal.pgph.0002201 (PMC10588837; doi:10.1371/journal.pgph.0002201)
Supplement: S1 Text — (DOCX) [file pgph.0002201.s003.docx]

S1 Text – Reflexivity Statement

1. How does this study address local needs and priorities

This study provides key insight which will inform the development of faith based health screening which makes best use of local assets relevant to health and well-being. Given recent predictions concerning the rise in rates of NCD in SSA, development of cost effective prevention strategies for NCD is an urgent priority. The findings of this study have produced practical recommendations which will promote culturally relevant approaches to the prevention of NCD in this context.

1. How were local researchers involved in study design

The study primary investigator for the lead institution in the UK was born in, and completed medical school in Ghana. A local primary investigator and several senior research staff were involved in the development of the funding proposal. All local research staff from Ghana supported the proposal and study to collect data which is being used to inform a future bid to pilot screening for NCD in the locality. Local senior researchers and research assistants helped the wider research team better understand the local setting and context, public health legislation and issues of access relating to uptake of screening services for NCD.

1. How has funding been used to support the research team

A stipulation of the funder was that 70% of the funding was to be spent in the LMIC. The funding has been used to support salary, research and infrastructure costs for the purchase of equipment.

1. How are research staff who collected data acknowledged

Staff who collected the data are acknowledged in the manuscript. Staff who were involved in data collection and analysis and who co-authored and or/edited the manuscript are included as co-authors

1. Do all members of the research partnership have access to study data

All researchers on the study have access to the data.

1. How was data used to develop analytical skills within the partnership?

Those who were interested were invited to workshops covering the fundamentals of realist evaluation and community based asset mapping techniques. Researchers from LMIC were also invited to attend sessions on academic writing provided by the lead institution.

1. How have research partners collaborated in interpreting study data?

Researchers from both sites had joint responsibility for generation of a coding framework and coding of data. Research partners reviewed the manuscript and provided feedback at all stages. Research findings and resources generated were shared with local public representatives and community leaders and refined based on feedback.

1. How were research partners supported to develop writing skills?
2. How will research products be shared to address local needs?

The findings of this study are being used to inform further grants for piloting of a screening education which will address the needs of local communities to improve access to screening services. Through this study we have identified many of the local resources and assets which already exist which can be leveraged to support implementation and maintenance of this programme.

1. How is the leadership, contribution and ownership of this work by LMIC researchers recognised within the authorship?

SC is included as joint first author on the manuscript. Colleagues in LMIC have first authorship of a further manuscript in preparation generated using additional data collected. The study local Principal Investigator based in Ghana is also included as a co-author

1. How have early career researchers across the partnership been included within the authorship team?

Early career researchers (AW, JA) were included within the authorship team. They were involved throughout the research and writing process.

1. How has gender balance been addressed within the authorship?

Seven authors are male, four are female. Two male researchers and two female researchers were principally responsible for the qualitative data analysis. However, we acknowledge that there could have been a better balance on the authorship team.

1. What safeguarding procedures were used to protect local study participants and researchers?

There were multiple safeguards and ethical approvals obtained and relevant training conducted with researchers to ensure confidentiality and privacy in regards to participant participation and data. Local researchers advised on cultural norms to be adhered to when visiting religious sites and when engaging with memebrs of local religious congregations
