# Supplementary material for: Cardiovascular disease prevention: Community Based Asset Mapping within religious networks in a rural Sub-Saharan African neighbourhood
Source: PLOS Glob Public Health. 2023 Oct 20;3(10):e0002201. doi: 10.1371/journal.pgph.0002201 (PMC10588837; doi:10.1371/journal.pgph.0002201)
Supplement: Attachment — Submitted filename: Response to reviewers.docx [file pgph.0002201.s004.docx]

Thank you for your helpful comments, we have responded to each and detailed the changes made below

1. Please include a complete copy of PLOS’ questionnaire on inclusivity in global research in your revised manuscript. Our policy for research in this area aims to improve transparency in the reporting of research performed outside of researchers’ own country or community. The policy applies to researchers who have travelled to a different country to conduct research, research with Indigenous populations or their lands, and research on cultural artefacts. The questionnaire can also be requested at the journal’s discretion for any other submissions, even if these conditions are not met.  Please find more information on the policy and a link to download a blank copy of the questionnaire here: https://journals.plos.org/globalpublichealth/s/best-practices-in-research-reporting. Please upload a completed version of your questionnaire as Supporting Information when you resubmit your manuscript.

This has been uploaded

2. Please send a completed 'Competing Interests' statement, including any COIs declared by your co-authors. If you have no competing interests to declare, please state "The authors have declared that no competing interests exist". Otherwise please declare all competing interests beginning with the statement "I have read the journal's policy and the authors of this manuscript have the following competing interests:"

I have read the journal's policy and the authors of this manuscript have the following competing interests: SS is in receipt of: Speaker honoraria from: AstraZeneca, Boehringer Ingelheim, Janssen, Lilly, MSD, Novo Nordisk, SB Communications, OmniaMed, Roche, Napp, NB Medical, Amgen, Advisory board honoraria from: AstraZeneca, Lilly, Boehringer Ingelheim, Janssen, MSD, Novo Nordisk, Takeda, Sanofi, Educational grants from: Boehringer Ingelheim, Lilly, Novo Nordisk, Takeda, Conference registration and subsistence from:, Boehringer Ingelheim, Janssen, Lilly, Novo Nordisk, Takeda.

All other authors have declared that no competing interests exist

3. Please amend your detailed Financial Disclosure statement. This is published with the article. It must therefore be completed in full sentences and contain the exact wording you wish to be published.

Included

c. If any authors received a salary from any of your funders, please state which authors and which funders.

N/A

Additional Editor Comments (if provided):

1. In the methods section it is not clear how the participants were selected in the study. It is mentioned that it was purposive selection. But the exact details of how they were selected in not provided.

Further detail on how participants were recruited has been included in the methods section

2. Why did the researchers choose to conduct the interviews and group discussions in the faith based organisations like churches and mosques? It would have been better to conduct them at the community level as even people from distances far away from the religious institutions could have participated and they could have brought in the perspective of distance.

The churches and mosques selected were located within the communities of interest (both rural and semi-rural) and were seen as the most accessible settings to carry out the IDIs and FGs and in line with what has worked to recruit previously. The chosen delivery method of faith based education and screening informed our decision to carry out FGs and IDIs within churches and mosques as we focussed discussions around this intended method. We have added detail into the methods section to justify the setting for FGs and IDIs

3. Who conducted the IDI and FGDs? What is their background? What religion did they practice? Some details of the interviewers would be helpful.

Further detail on the background of IDI and FG facilitors has been added

4. A reflexivity note by the interviewers would also be helpful to interpret the findings of the research.
5. More details must be provided on how the data analysis was done. how many researchers did the coding and theme building? what processes were adopted for coding and theorising?

This has been added to the methods section

6. The entire paper lacks any verbatim quotes. The authors must provide verbatim quotes along with a brief description of the persons who said those quotes.

Verbatim quotes are provided in table 2 and 3, together with brief (anonymised) details on the person who the quote was attributed to. This table will be embedded within the results section in the published paper.

7. There are several methods used by researchers to get participants to engage actively in participatory methods. For example, they may be asked to draw maps, they may be asked to sort resources into groups, etc. What participatory action method was used by the authors in this study? This is not explained.

The majority of data collection for the asset mapping took place through face to face discussion via FGs and IDIs, whilst we did not deploy arts-based methods (drawing maps, photo-voice etc) we used an iterative process of data collection, reflection and feedback, with FG and IDI and workshop participants to co-produce the ‘asset map’ (figure 1) and to co-produce and rank the list of screening recommendations. Further detail on this has been included in the methods (data collection) section.

8. There is no mention of what methods were adopted to ensure credibility, dependability, transferability and confirmability of the qualitative research findings. This must be clarified in the paper. the authors should carefully go through these critical inputs and revise their manuscript accordingly.

Further detail has been provided within the methods section, and as part of the reflexivity statement.

5. Review Comments to the Author

Please use the space provided to explain your answers to the questions above. You may also include additional comments for the author, including concerns about dual publication, research ethics, or publication ethics. (Please upload your review as an attachment if it exceeds 20,000 characters)

Reviewer #1: Comments and Suggestions for Authors
The authors conducted a study to identify and catalogue community-based assets for the purposes of developing and deploying a screening and education programme for cardiometabolic risk factors (diabetes and hypertension) within religious organisations in a local community in a rural Ghanaian context. The paper is very well-written, well-organized, and interesting. They used appropriate participatory approaches and provided an adequate discussion of the results. I have a few minor editorial comments that will hopefully improve the paper.

* The author has mentioned that they have used Community-based Asset Mapping with a different approach. Did the authors consider ways that findings from different stakeholders traditional (faith healers, traditional healers) and modern medicine (nurses, pharmacists, doctors) can be applied in best practice for CVD screening and educational programs?

We acknowledge that the relationship between traditional and modern medicine in this context is complex and nuanced and highly relevant to our focus on health screening and education uptake. Whilst we have attempted to briefly touch upon some of these issues, the manuscript we have submitted is limited to presentation of the results from the asset mapping exercise and linked recommendations for the deployment of screening services within this context. We are in the process of preparing a second manuscript which presents additional qualitative data relating to health beliefs and attitudes to screening which covers the issue raised by the reviewer, in more detail.
